# Blue Marble Health: An Innovative Plan to Fight Diseases of the Poor amid Wealth

**DOI:** 10.3201/eid2302.161801

**Published:** 2017-02

**Authors:** John Iskander

**Affiliations:** Centers for Disease Control and Prevention, Atlanta, Georgia, USA

**Keywords:** neglected tropical diseases, global health, parasites, bacteria, viruses, vector-borne infections, helminths, protozoa

Neglected tropical diseases (NTDs) comprise 17 helminthic, protozoan, bacterial, and vectorborne viral diseases that disproportionately affect the world’s poor (*1*). This diverse group of infections and infestations includes hookworms, leishmaniasis, Chagas disease, dengue fever, and trachoma. In Blue Marble Health, Dr. Peter Hotez, dean of the National School of Tropical Medicine and founding editor of PLoS Neglected Tropical Diseases, shifts the focus of global health from a traditional developed versus developing world paradigm toward impoverished populations living amid wealthy countries, who suffer heavily from NTDs. The book’s title invokes an iconic image of the earth as seen from space by the Apollo astronauts.

On the basis of previous work, Dr. Hotez asserts that nearly every person in poverty is infected with at least 1 NTD, “the most important diseases you’ve never heard of.” Blue Marble Health contains helpful summaries of 11 major NTDs in Chapter Two. The main body of the text is organized according to the book’s geographic focus on the G20 countries (the world’s major economies) plus Nigeria. These featured countries account for half of the world’s NTDs. It might surprise many to learn that 12 million US residents live with a neglected parasitic infection. Despite this widespread burden of disease, only 0.003% of world gross domestic product is spent on NTD research.

The book’s readability is enhanced by its reasonable length and its organization according to geography. Although the lack of detailed attention to zoonotic transmission issues is surprising, the book addresses broader forces driving NTD emergence, including co-morbidity with noncommunicable diseases, climate change, and regional conflict. Timely content includes discussion of the West Africa Ebola epidemic and the emergence of Zika virus in the context of NTDs.

The book contains copious figures and tables, which are just as likely to focus on economic indicators as on direct measures of NTDs. Minor formatting issues detract from the book’s readability. For example, magazine-style text inserts crowd the layout at times. Because the field of global health is replete with acronyms and initialisms, a glossary would have been a helpful addition. Although many of the book’s references come from the journal the author edits, these are balanced by citations from other sources.

Much of the book’s focus on interventions to combat NTDs concerns wider use of mass drug administration campaigns. However, as president of the Sabin Vaccine Institute, Hotez briefly describes vaccines for hookworm disease and schistosomiasis that are currently in clinical trials. Hotez’ enthusiasm for and deep knowledge about NTDs comes through clearly. Drawing on his experience as a US Science Envoy, the author clearly wants his book to be read and acted on by the leaders of G20 countries. His ideas for multinational policy solutions include the need for increased surveillance along with local research and development investment.

The intended audience for this book goes well beyond public health and tropical medicine specialists. The policy-heavy content and clear advocacy tone make it appropriate for students and teachers of global health, public policy, and foreign affairs. The book should also be of value to persons interested in social determinants of health, health economists, and historians of public health and international development.
